# Systematized and efficient: organization of critical care in the future

**DOI:** 10.1186/s13054-022-04244-1

**Published:** 2022-11-28

**Authors:** Annette M. Esper, Yaseen M. Arabi, Maurizio Cecconi, Bin Du, Evangelos J. Giamarellos-Bourboulis, Nicole Juffermans, Flavia Machado, Sandra Peake, Jason Phua, Kathryn Rowan, Gee Young Suh, Greg S. Martin

**Affiliations:** 1 Division of Pulmonary, Allergy, Critical Care and Sleep Medicine, Emory University and Grady Memorial Hospital, Atlanta, Georgia USA; 2 Intensive Care Department, Ministry of the National Guard Health Affairs, King Abdulaziz Medical City, King Saud Bin Abdulaziz University for Health Sciences, King Abdullah International Medical Research Center, Riyadh, Saudi Arabia; 3 Department of Anaesthesia and Intensive Care, Humanitas University, Milan, Italy; 4 National Key Laboratory of Rare, Complex and Critical Diseases, Medical ICU, Union Medical College Hospital, Peking/Beijing, China; 5 4th Department of Internal Medicine, National and Kapodistrian University of Athens, Medical School, Athens, Greece; 6 Laboratory of Translational Intensive Care Erasmus Medical Center, Rotterdam, the Netherlands; 7 OLVG Hospital, Amsterdam, the Netherlands; 8 Anesthesiology, Pain and Intensive Care Department, Hospital São Paulo, Federal University of São Paulo, São Paulo, Brazil; 9 Department of Intensive Care Medicine, The Queen Elizabeth Hospital, Faculty of Health and Medical Sciences, University of Adelaide, Adelaide, Australia; 10 Faculty of Medicine, Nursing and Health Sciences, Monash University, Melbourne, Australia; 11 FAST and Chronic Programmes, Alexandra Hospital, Singapore, Singapore; 12 Division of Respiratory and Critical Care Medicine, National University Hospital, National University Health System, Singapore, Singapore; 13 Intensive Care National Audit and Research Centre, London, UK; 14 Samsung Medical Center, Sungkyunkwan University School of Medicine, Seoul, Korea

**Keywords:** Critical care, Intensive care medicine, ICU space

## Abstract

Since the advent of critical care in the twentieth century, the core elements that are the foundation for critical care systems, namely to care for critically ill and injured patients and to save lives, have evolved enormously. The past half-century has seen dramatic advancements in diagnostic, organ support, and treatment modalities in critical care, with further improvements now needed to achieve personalized critical care of the highest quality. For critical care to be even higher quality in the future, advancements in the following areas are key: the physical ICU space; the people that care for critically ill patients; the equipment and technologies; the information systems and data; and the research systems that impact critically ill patients and families. With acutely and critically ill patients and their families as the absolute focal point, advancements across these areas will hopefully transform care and outcomes over the coming years.

## Introduction

The origins of critical care medicine are rooted in organization, with Florence Nightingale aggregating the sickest patients into special areas where more critically ill and injured individuals could be cared for and closely monitored. Over time, critical care infrastructure has evolved to incorporate multidisciplinary personnel, in addition to a variety of advanced technologies and resources. Critical care as we know it today is complex and remains challenging in many ways. The heterogeneity of underlying illnesses that require critical care adds to the complexity. While the care of the critically ill patient has evolved, there remain many challenges including cost of critical care, access to/appropriate use of critical care beds, staffing limitations, and variation in management strategies. Most recently, the COVID pandemic has placed a significant strain on healthcare systems overall and highlighted some of the obstacles that we continue to face in critical care despite all the technological advancements. Furthermore, the emotional environment of the intensive care unit (ICU) was on full display, and we were reminded of the vulnerability of patients and providers. This emphasizes the need to think outside the box when conceptualizing future directions for models of critical care medicine [1].

In this commentary, we present a vision, based on diverse experiences, personal opinions, and foundational prior evidence, of the organization of critical care in the years to come. As we conceive the future of critical care, the patient remains the focal point, and therefore, strategies that result in better and more personalized care for the critically ill should be implemented. For critical care to be effective in the future, there are opportunities to enhance multiple facets of critical care, including the physical ICU space, the people that care for ICU patients, ICU equipment and technologies, ICU information systems, and the systems for research in the critically ill (see Table 1). The ICU of the future should be designed and organized with all these facets in mind to optimize outcomes, with emphasis on initiatives that may potentially impact pre- and post-care of the critically ill.

**Table 1:** The integral parts of the future intensive care unit

*ICU Space*
Private rooms with maximum healthcare functionality
Rooms that replicate benefits of the home environment
Improved visibility between patients and providers
Bedside nursing station
Proximity to key hospital resources (e.g., radiology)
Improved access for families
Virtual access to ICU patients and providers
Critical care outside the ICU
*ICU Team*
Intensivist physicians
Advanced practice providers
Nurses
Pharmacists
Dieticians
Physical and occupational therapists
Speech therapists
Social workers
Family members and caregivers
Providing critical care outside the ICU
*ICU Equipment*
Wireless and wearable sensors
Noninvasive methods for physiologic and biologic monitoring
Patient beds that prevent injury and facilitate rehabilitation
Advances in organ support equipment *
Systems biology point-of-care devices
*ICU Systems*
Provider-oriented EMR systems that facilitate patient care
Seamless medical device integration
Encrypted sharing and enhanced cybersecurity
Effortless data retrieval and reporting
Incorporation of AI and machine learning
Policies facilitated by protocols
*ICU Research*
Focus on humanity and equity in ICU care
Characterize individual trajectory throughout critical illness
Deliver clinical application of omics
Incorporation of mixed methods approaches
Routine use of adaptive trial designs
Utilizing EHR and clinical information systems for conducting research
Development of collaborative global research networks

*Examples include invasive and noninvasive ventilation, renal replacement, extracorporeal liver support, cardiovascular support modalities, blood purification technologies

### Physical ICU space

The components of the physical ICU space are essential to the care of the critically ill, and there are opportunities for novel designs that may improve outcomes. In the future, critically ill patients will be managed in individual ICU rooms that are designed to maximize functionality, maintain privacy, promote healing in a humanized environment, facilitate infection control, contribute to patient safety and improve communication [2–4]. These individual room structures should be such that they could allow the management of multiple patients or facilitate the conversion to an open space if required at times of high-volume patient needs (e.g., disasters and epidemics). The ICU layout will enable effective care delivery, as the room distribution allows nurses and other healthcare providers to maintain visibility and easy access to multiple patients at the same time [5]. Visibility of patients will be maintained through smart glass transparent walls, doors and windows that can be opacified when needed. The nursing station will be at the bedside, so the ICU staff are closer to patient care. The location of the ICU and the design will consider the requirements of daily workflows [6], such that the proximity to the emergency department, operating rooms, blood bank and radiology departments will facilitate efficient and safe patient transfers.

The rooms will simulate home environments, with their décor, furnishings and natural light to minimize delirium and using windows to reveal or simulate a healing natural scene or garden [7, 8]. Noise will be kept at bedroom level and exposure to daylight will preserve the diurnal rhythm and prevent delirium [9]. The patient and caregivers will have control over their environment, including steering the bed to face the window to the outside, management of light and temperature levels, accessing entertainment and educational resources, and virtually connecting with family. The multifunctional ICU beds will combine the comfort of home with the functionality required for patient care. Early rehabilitation of ICU patients will be secured with the availability of a specialized gymnastic facility. There will be different kinds of step-down or intermediate care facilities close to the ICU. A long-term acute care facility will provide care for patients with prolonged weaning largely managed by physician assistants. Ceiling or wall-mounted monitoring systems will allow mobility and easy access to the patient in case of emergencies.

The structure and access to the ICU will facilitate the unrestricted presence of family members, who will no longer be considered visitors, but rather an integral part of the healing process. Facilities for the family stay will be easily accessible and designed to help meet the needs of families under stress. Family members and care providers may be virtually available by utilizing cameras and holographic systems [10]. Each ICU will have a staff lounge and facilities to support wellness, education and productivity.

### Expanding ICU care beyond its walls

Critical care in the future will not be confined to being within the walls of an ICU [11]. Policymakers, healthcare administrators and clinical teams in ICUs and beyond will work together to facilitate pre-hospital critical care treatment in the home and the ambulance, and the early detection of and rapid response to deterioration in the emergency room and the general ward [12, 13]. Therefore, critical care will no longer be confined to the walls of the ICU, as many patients on the wards will be monitored using wearable devices, and the hospital can be viewed as a large ICU with individualized levels of care intensity. Each ICU clinician must be aware of the high prevalence of the potentially life-changing post-intensive care syndrome, with its adverse impact on the physical, cognitive and mental health of ICU survivors [14]. Post-ICU follow-up in the form of comprehensive medical care, physical therapy, psychological and emotional support will be part of the continuum of post-ICU care. Care for family members who are at a high risk of physical exhaustion, post-traumatic stress disorder, anxiety and depression, will become routine [15].

### People who care for ICU patients

It has been projected that the intensivist workforce will decline in the future [16], requiring more of a focus on interprofessional care models. Current and future best practice for critically ill patients requires a coordinated, team-based, multidisciplinary approach involving specialized medical and nursing staff supported by allied health clinicians such as dieticians, pharmacists, physical and occupational therapists, speech therapists and social workers. Emerging evidence that early mobilization of mechanically ventilated patients may reduce ICU-acquired weakness and increase the number of ventilator-free days and discharge to home rate will necessitate a greater emphasis on patients that are more awake and more mobile with dedicated, physiotherapy-led, mobility teams that specialize in early rehabilitation for the critically ill [17].

Traditionally, the ICU team has focused on those patients present within the ICU. However, there is increasing demand for ICU services across the whole of hospital with rapid response teams and 24-h in-hospital medical specialist staffing models now commonplace in many institutions. Community expectations, technological advances, increasing patient age, co-morbidities and frailty have also increased the demand for ICU services. A great emphasis will be placed on the early detection and management of critical illness.

Because attrition of the ICU workforce, including physicians and other professionals will remain a challenge, ensuring a sustainable workforce for the future will require both increased importance on staff health and welfare to mitigate burnout [18] and the adoption of staffing models that reflect changing societal attitudes to the working environment e.g., flexible working hours and training requirements, job-sharing, workload burden and diversity, including greater female representation amongst medical specialists [19]. We must continue to move away from the hierarchical structure of medical teams and toward a collaborative environment. In addition to traditional training, team-based learning with simulation will improve the synergy of the ICU team. The role of administrative leadership working with frontline staff to create a vision and strategy to this end will be of the utmost importance [20].

Elements discussed elsewhere in this commentary will also influence the ICU workforce, and ICU clinicians of the future will need to be adept in embracing digital and technological advances, data science and artificial intelligence.

There is increasing recognition that families play an integral role in the ICU, not only in day-to-day patient management during acute illness but also in end-of-life care [21]. The benefits of fostering meaningful engagement include emotional and spiritual support for patients and families, informed clinical decision making that reflects the patient’s beliefs and wishes and assistance in the recovery phase, particularly post-ICU. This will necessitate that members of the ICU team cultivate communication skills not only with their colleagues, but with patients and their families. Producing compassionate, empathic, and ethically and culturally sensitive clinicians that are resilient to the many challenges of caring for critically ill and dying patients requires inter-professional training programs that extend beyond teaching the core ICU management skills to ensure holistic, patient-centered, personalized care remains the focus.

### Equipment in the ICU

The physical and professional aspects of critical care have historically been influenced by the equipment used to support patients and their failing organs. Technical innovations will bring remarkable changes in the equipment in the ICU. Bedside monitors may allow direct visualization of patient status (such as vital signs, electrolytes, gas exchange and hemodynamics) based on physiologic information from wireless and wearable sensors, with most parameters measured in noninvasively, thus reducing the need of phlebotomy. For example, (a) real-time arterial pressure waveform can be captured from the radial artery or the carotid artery by means of the volume clamp method or applanation tonometry [22, 23], with the potential to estimate ventricular dysfunction and fluid overload using pulse contour analysis; and (b) multimodal probes and sensors, hemodynamic monitoring can go far beyond global physiologic parameters, but include regional perfusion (i.e., sublingual microcirculation, cerebral perfusion) and oxygenation, or even metabolic monitoring (electrolytes and lactate in skin fluid).

New devices for organ support will emerge, from evolution of existing technologies to novel ways to support failing organs, such as the brain. Mechanical ventilators, albeit much smaller, can display important physiologic parameters crucial to the development of ventilator-induced lung injury, such as asynchrony index, transpulmonary pressure, mechanical power and the extent of lung inhomogeneity [24, 25]. Technical advances in bioengineering will reshape blood purification. These include wearable artificial kidney, which enables continuous dialysis, requires adequate vascular access, antithrombogenic circuit with minimum priming volume, small-sized dialyzer and capability of remote control [26] or a bioartificial kidney implant with hemofilter made up of silicon semiconductor membranes that remove waste products from blood and a bioreactor containing renal tubule cells that regulate water volume, electrolyte balance and other metabolic functions [27]. In addition, extracorporeal magnetic separation-based blood purification will allow increased water and solute removal, including rapid and selective removal of disease-causing compounds from whole blood [28].

Perhaps most important, traditional biochemistry will be replaced by point-of-care devices using the integration of gene expression protein biomarkers and metabolites to provide more personalized critical care. Point-of-care testing will characterize the relevant biomarkers, such as the immune state, endothelial function and coagulation system, to endotype individual patients in real time to optimize treatments such as antibiotics, vasopressors and immunotherapies. Repeat measurements of these biomarkers will suggest how treatment needs to be escalated or de-escalated and how liver and kidney function risk to become deteriorated [29]. All drug concentrations will be measured by microfluidic devices, and they will be connected to infusion pumps to adjust the administered dose of drugs. As novel devices and equipment for use in the ICU are developed, it will be important to consider ways to maintain cost-effective critical care and ensure accessibility.

### Systems that underlie ICU care

Over the next 25 years, health information technology will be far more advanced, widely available and ubiquitously deployed, even in today’s low- and middle-income countries. While electronic medical record (EMR) systems will be the norm, expectations that their use may reduce mortality, length of stay, and costs have to date not been fulfilled [30]. To fully reap their potential benefits, concerted efforts in value-driven design by ICU clinicians and systems engineers are required. First, EMR systems will minimize time spent by busy healthcare workers on documentation—which is hardly the case now—by focusing on essential data fields and enabling automatic data capture. Second, medical device integration solutions will synchronize data from various clinical equipment and monitors to the EMRs, and in so doing facilitate efficient and accurate data transfer and eliminate the panoply of integrating devices and systems. Third, an emphasis on cybersecurity is key, especially as geopolitics will become increasingly complex and cyber-terrorists will become more sophisticated over time. Fourth, easy retrieval and analysis of data from EMRs for audits, benchmarking, quality improvement and research are important [31]. Finally, artificial intelligence and machine learning will allow prediction of clinical trends and provide real-time decision support [32].

Policies both administrative and clinical of the ICU of the future should be facilitated by protocols. This notwithstanding, it is noteworthy that the mere presence or the sheer number of protocols has not been associated with compliance by staff and outcomes for patients [33]. Several qualities are thus needed for protocols to work. First, they must be based on sound evidence, examples being protocols for ventilator weaning, patient sedation, sepsis treatment and pandemic readiness [34–36]. Second, multi-faceted efforts to improve compliance to them are required, including design of steps to maximize ease of use and education of staff on the science behind these steps. Third, ICU teams should recognize the inherent rigidity of protocols and allow leeway for clinician judgment in the upcoming era of personalized medicine.

### Research in critical care medicine

Evidence from research will continue to play a central role in our understanding of critical illness and in informing the organization and delivery of high-quality critical care over the next 25 years, and beyond. Establishing what is quality critical care, through research, will continue with an expanded scope, beyond considering solely the effectiveness of care, increasingly, to considering the importance of the humanity of care (care delivered with respect and dignity) and the equity of care (care equally accessible to all)—both important wider elements of high-quality critical care. Evaluating all these elements will extend the traditional focus of our research and further inform treatment for individual patients.

Continuing to expand the scope of research into critical illness and the associated care will lead to greater consideration of the full trajectory of critical illness for a person—starting/ending in the community. Continued evaluation of patients along this trajectory will, necessarily, encompass both susceptibility to, and full recovery from, critical illness and expand our consideration and use of broader patient-centered outcomes beyond mortality. The boundaries of consideration of susceptibility to critical illness will emphasize the emerging and important role of omics (e.g., genomics, epigenomics, transcriptomics, proteomics and metabolomics) to our knowledge base—each of these areas potentially offering the possibility to understand and view biology in a way previously unthinkable. Using omics for better identification of more homogeneous critically ill patients from within our broad syndromic definitions, with test results available in real-enough-time for research participation, will enhance patient selection. The resulting prognostic enrichment will allow the identification of specific phenotypes that may benefit from selected interventions, improving the odds for the identification of therapies that could lead to better outcomes instead of the current scenario of negative trials conducted in heterogeneous groups of patients. Technical innovations will also provide better tools for hemodynamic and ventilation strategies resulting in more standardized interventions feasible to be used or tested in clinical trials.

While randomized clinical trials will remain the mainstay for evaluation, other rigorous, mixed methods approach (both quantitative and qualitative) will be increasingly needed for comprehensive evaluation of the organization and delivery of care for the critically ill. The voice of the person (patient/public) will hopefully become even more apparent with increased active involvement and engagement. As a consequence, critical care researchers will increasingly use patient-centered outcomes, less focused on survival and physiologic impairment and prioritizing outcomes such as functional status and quality of life. With regard to randomized clinical trials (RCTs), the evolution of platforms (evaluating more than one intervention simultaneously) and adaptive approaches to design and analysis will continue to move us in the direction of research in critical care becoming embedded in clinical practice and facilitate the evolution of learning healthcare systems [37]. Research platforms will also allow for seamless phase II to phase III RCTs, improving the pipeline for evaluation.

Available data will be more efficiently used, and will continue to provide the infrastructure for research on critical illness. With the wider introduction of digital platforms (electronic health records/clinical information systems) across healthcare systems and improvements in the capture of accurate (complete, valid and reliable) data, the degree of manual data collection will reduce alleviating the burden and costs of conducting research. With wider introduction of individual patient identifiers into healthcare systems and, with appropriate governance in place, the ability to link data across databases for the same patient will be enhanced as will the research needed to understand the full trajectory of critical illness. Transparent, open, secure access to linked accurate data will facilitate exploration and the potential generation of improved learning from machines, for example, artificial intelligence [38].

Regional and national research networks have been the bedrock of critical care research. In the recent COVID-19 pandemic, the realization of global research networks and platforms has occurred. The model of global working including both high- and low-resourced settings, should potentially improve equality, diversity and inclusion both for researchers and for research participants—with emphasis on doing the research where there is most need. Avoiding exploitative research in which high-income countries’ researchers do not acknowledge properly their local partners and collaborators from less known or resourced settings is a key step to achieve equality. This should not be limited to adequate authorship, but also consider the prioritization of relevant local research questions, with their respective funding, and contribution to improvement in local research capacity which includes participation from the concept of the study, the conduction, the interpretation of data and writing. But the challenges of ensuring that all voices are heard, and all researchers acknowledged will need to be addressed—as will the current incentives for research advancement. Identifying new ways to recognize and measure impact of research, continuing to move away from authorship and grant-holding, will be important for the global democratization of critical care research as will free, open-access, opportunities for research evidence dissemination.

## Conclusion

Although critical care has made significant advances over the years, the substantial heterogeneity of critical care illnesses has made it a challenge to make sufficient progress with respect to therapeutics. In order for progress to continue, the infrastructure of critical care needs to be transformed and the focus must pivot to ways that enhance personalized medicine. Technology will continue to make strides which should provide us with the tools for redesigning processes of critical care management. It is imperative that we shift the paradigm and develop models of care that allow for the care of the critically ill beyond the walls of the ICU. The global variability in many aspects of critical care will forever remain, yet working to achieve this future vision for critical care organization will lead to higher-quality care across systems and continents, including more humanistic and more personalized care with fewer errors and better quality, as the goal will always remain to provide the highest quality patient care and outcomes.

## Data Availability

Not applicable.

## References

[1] Maslove DM, Tang B, Shankar-Hari M (2022). Redefining critical illness. Nat Med.

[2] Halpern NA (2014). Innovative designs for the smart ICU: part 2: the ICU. Chest.

[3] Sundberg F, Fridh I, Lindahl B, Kareholt I (2021). Associations between healthcare environment design and adverse events in intensive care unit. Nurs Crit Care.

[4] Caruso P, Guardian L, Tiengo T, Dos Santos LS, Junior PM (2014). ICU architectural design affects the delirium prevalence: a comparison between single-bed and multibed rooms*. Crit Care Med.

[5] Lu Y, Ossmann MM, Leaf DE, Factor PH (2014). Patient visibility and ICU mortality: a conceptual replication. HERD.

[6] Thompson DR, Hamilton DK, Cadenhead CD (2012). Guidelines for intensive care unit design. Crit Care Med.

[7] Sundberg F, Fridh I, Lindahl B, Kareholt I (2021). Visitor's experiences of an evidence-based designed healthcare environment in an intensive care unit. HERD.

[8] Verderber S, Gray S, Suresh-Kumar S, Kercz D, Parshuram C (2021). Intensive care unit built environments: a comprehensive literature review (2005–2020). HERD.

[9] Luetz A, Grunow JJ, Morgeli R (2019). Innovative ICU solutions to prevent and reduce delirium and post-intensive care unit syndrome. Semin Respir Crit Care Med.

[10] Halpern NA, Anderson DC, Kesecioglu J (2017). ICU design in 2050: looking into the crystal ball!. Intensive Care Med.

[11] Hillman K (2002). Critical care without walls. Curr Opin Crit Care.

[12] Seymour CW, Rea TD, Kahn JM, Walkey AJ, Yealy DM, Angus DC (2012). Severe sepsis in pre-hospital emergency care: analysis of incidence, care, and outcome. Am J Respir Crit Care Med.

[13] Maharaj R, Raffaele I, Wendon J (2015). Rapid response systems: a systematic review and meta-analysis. Crit Care.

[14] Needham DM, Davidson J, Cohen H (2012). Improving long-term outcomes after discharge from intensive care unit: report from a stakeholders' conference. Crit Care Med.

[15] Azoulay E, Resche-Rigon M, Megarbane B (2022). Association of COVID-19 acute respiratory distress syndrome with symptoms of posttraumatic stress disorder in family members after ICU discharge. JAMA.

[16] Angus DC, Kelley MA, Schmitz RJ (2000). Caring for the critically ill patient. Current and projected workforce requirements for care of the critically ill and patients with pulmonary disease: can we meet the requirements of an aging population?. JAMA.

[17] Zhang L, Hu W, Cai Z (2019). Early mobilization of critically ill patients in the intensive care unit: a systematic review and meta-analysis. PLoS One.

[18] Kerlin MP, McPeake J, Mikkelsen ME (2020). Burnout and joy in the profession of critical care medicine. Crit Care.

[19] Venkatesh B, Mehta S, Angus DC (2018). Women in intensive care study: a preliminary assessment of international data on female representation in the ICU physician workforce, leadership and academic positions. Crit Care.

[20] Roy K, Brunet F (2005). The role of leadership in overcoming staff turnover in critical care. Crit Care.

[21] Burns KEA, Misak C, Herridge M (2018). Patient and family engagement in the ICU. Untapped opportunities and underrecognized challenges. Am J Respir Crit Care Med.

[22] Michard F (2016). Hemodynamic monitoring in the era of digital health. Ann Intensive Care.

[23] Michard F, Pinsky MR, Vincent JL (2017). Intensive care medicine in 2050: NEWS for hemodynamic monitoring. Intensive Care Med.

[24] Kacmarek RM (2011). The mechanical ventilator: past, present, and future. Respir Care.

[25] Gattinoni L, Marini JJ, Collino F (2017). The future of mechanical ventilation: lessons from the present and the past. Crit Care.

[26] Chan CT, Covic A, Craig JC (2013). Novel techniques and innovation in blood purification: a clinical update from kidney disease: improving global outcomes. Kidney Int.

[27] D K. This bioartificial organ could one day save ‘millions’ living with kidney disorders. 2021.

[28] Herrmann IK, Schlegel AA, Graf R, Stark WJ, Beck-Schimmer B (2015). Magnetic separation-based blood purification: a promising new approach for the removal of disease-causing compounds?. J Nanobiotechnology.

[29] Sweeney TE, Liesenfeld O, Wacker J (2021). Validation of inflammopathic, adaptive, and coagulopathic sepsis endotypes in coronavirus disease 2019. Crit Care Med.

[30] Thompson G, O'Horo JC, Pickering BW, Herasevich V (2015). Impact of the electronic medical record on mortality, length of stay, and cost in the hospital and ICU: a systematic review and metaanalysis. Crit Care Med.

[31] Higgins TL, Freeseman-Freeman L, Stark MM, Henson KN (2022). Benchmarking inpatient mortality using electronic medical record data: a retrospective, multicenter analytical observational study. Crit Care Med.

[32] Martin GS (2019). The intersection of big data, artificial intelligence, precision and predictive medicine to create the future of critical care. ICU Manag Pract.

[33] Sevransky JE, Checkley W, Herrera P (2015). Protocols and hospital mortality in critically Ill patients: the united states critical illness and injury trials group critical illness outcomes study. Crit Care Med.

[34] Pun BT, Balas MC, Barnes-Daly MA (2019). Caring for critically Ill patients with the ABCDEF bundle: results of the ICU liberation collaborative in over 15,000 adults. Crit Care Med.

[35] Levy MM, Rhodes A, Phillips GS (2014). Surviving Sepsis Campaign: association between performance metrics and outcomes in a 7.5-year study. Intensive Care Med.

[36] Arabi YM, Azoulay E, Al-Dorzi HM (2021). How the COVID-19 pandemic will change the future of critical care. Intensive Care Med.

[37] Granholm A, Alhazzani W, Derde LPG (2022). Randomised clinical trials in critical care: past, present and future. Intensive Care Med.

[38] Yoon JH, Pinsky MR, Clermont G (2022). Artificial intelligence in critical care medicine. Crit Care.

